# Supplementary material for: Design, Synthesis and Bioactive Evaluation of Oxime Derivatives of Dehydrocholic Acid as Anti-Hepatitis B Virus Agents
Source: Molecules. 2020 Jul 24;25(15):3359. doi: 10.3390/molecules25153359 (PMC7435646; doi:10.3390/molecules25153359)
Supplement: Supplementary file 1 [file molecules-25-03359-s001.pdf]

Design, Synthesis and Bioactive Evaluation of Oxime Derivatives of Dehydrocholic Acid as Anti-Hepatitis B Virus Agents

Zhuocai Wei[1,†], Jie Tan[2, 1†], Xinhua Cui[1], Min Zhou[1], Yunhou Huang[1], Ning Zang[3], Zhaoni Chen[4], Wanxing Wei[1,*]

Table 1 Cytotoxicity and inhibitory effect of target compounds on HBeAg and HBsAg *in vitro*

Compd	CC_{50}[a] (μM)	HBeAg [d]		HBsAg [e]	
		IC_{50}[b] (μM)	SI[c]	IC_{50}[b] (μM)	SI[c]
2a-1	377.88 ± 25.31**	229.34 ± 12.78	1.65	630.32 ± 34.95**	0.60
2a-2	210.69 ± 17.11**	248.66 ± 47.61	0.85	-[f]	-
2a-3	169.10 ± 5.75**	187.76 ± 9.51	0.90	-	-
2b-1	>1000**	96.64 ± 28.99**	10.35	-	-
2b-2	728.15 ± 45.22*	-	-	-	-
2b-3	544.73 ± 28.92	49.39 ± 12.78**	11.03	-	-
2c-1	155.05 ± 30.83**	110.61 ± 28.30**	1.40	300.00 ± 15.30**	0.52
2c-2	101.04 ± 10.66**	151.23 ± 32.11*	0.67	464.29 ± 20.10**	0.22
2c-3	90.85 ± 15.59**	105.19 ± 22.20**	0.86	180.57 ± 52.83	0.50
0-1	>1000**	-	-	-	-
0-2	470.47 ± 6.35*	119.03 ± 1.86**	3.95	-	-
0-3	>1000**	-	-	-	-
DHCD [g]	>1000**	-	-	-	-
ETV [h]	600.12 ± 23.44	246.87 ± 50.03	2.43	161.24 ± 35.94	3.72

[a] CC_{50} is 50% cytotoxicity concentration in HepG 2.2.15 cells; [b] IC_{50} is 50% inhibitory concentration; [c] SI (selectivity index) = CC_{50}/IC_{50}; [d] HBeAg: hepatitis B e antigen; [e] HBsAg: hepatitis B surface antigen; [f] The inhibition ratio less than 50% in the test concentration range; [g] Dehydrocholic acid (DHCA) is the raw material of reaction; [h] Entecavir (ETV) as the positive control. Data were expressed as mean ± S.D. (n = 3). * Compared with the positive control index: $p < 0.05$. ** Compared with the positive control index: $p < 0.01$.

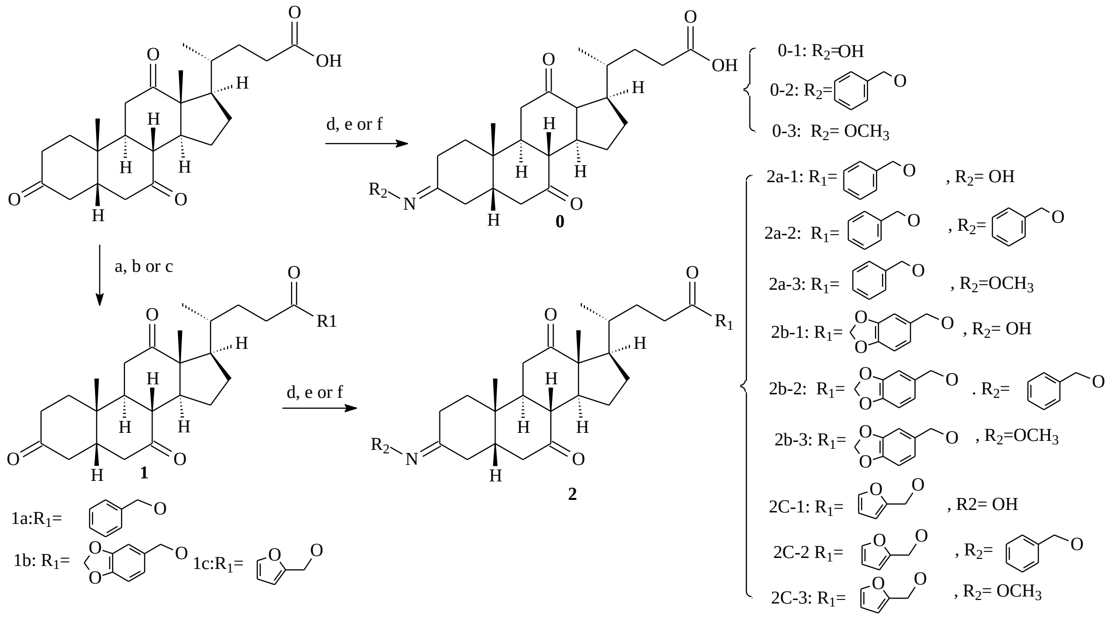

Scheme 1. Synthetic routes of target compounds

Reagents and conditions: (a) benzyl alcohol, DMAP, DCC/CH_2Cl_2, 0.5 h, 0 ºC; overnight, rt; (b) piperonyl alcohol, DMAP, DCC/CH_2Cl_2, 0.5 h, 0 ºC; overnight, rt; (c) furfuryl alcohol, DMAP, DCC/CH_2Cl_2, 0.5 h, 0 ºC; overnight, rt; (d) $NH_2OH\cdot HCl$, Sodium acetate trihydrate/CH_2Cl_2, reflux, 3~12 h; (e) $NH_2OCH_2C_6H_5\cdot HCl$, Sodium acetate trihydrate/CH_2Cl_2, reflux, 3~12 h; (f) $NH_2OCH_3\cdot HCl$, Sodium acetate trihydrate/DCM, reflux, 3~12 h.

Table 2. Docking results of target compounds with bile acid receptor

Compound	S(kcal/mol)	H-bonds	Distance（Å）	Residue	Atoms Involved
2a-1	-11.99	1	2.69	Arg 395	O of N-OH
2a-2	-13.07	2	2.74	His 445	O of C_{12}=O
			2.08	Gln 396	O of C_7=O
2a-3	-10.85	-	-	-	-
2b-1	-13.46	3	3.54	Tyr 397	O of Piperony
			2.85	Gln 396	
			2.92	His 447	O of C_{12}=O
2b-2	-12.88	3	3.07	Tyr 397	O of Piperony
			2.18	Lys 321	
			2.81	Arg 395	N of C=N-O
2b-3	-12.14	4	2.68	Arg 686	O of Piperony
			3.36	Tyr 397	O of Piperony
			3.00	Ser 392	O of C_{24}=O
			2.83	Asn 444	O of C_{12}=O
2c-1	-12.46	1	1.85	Arg 395	O of N-OH
2c-2	-12.03	2	2.15	Arg 441	O of C_7=O
			2.65		
2c-3	-11.60	-	-	-	
0-1	-11.71	3	2.70	Ser 392	O of N-OH
			2.70	Ser 392	O of N-OH
			2.50	Trp 469	O of C_{24}=O
0-2	-12. 49	2	3.02	Trp 469	H of C_{24}-OH
			2.41	Asp 470	
0-3	-11.51	2	2.26	Gln 396	O of C_{12}=O
			2.23	Gly 322	H of C_{24}-OH
Cholic acid	-12.35	8	2.64	Asp 394	H of C_{24}-OH
			3.62		
			2.55	Arg 441	O of C_{24}-OH
			1.68	Gly 322	H of C_7-OH
			3.30	Lys 321	
			3.54	Ile 468	H of C_3-OH
			1.30	Asp 470	
			2.91	Lys 321	O of C_3-OH
DHCA	-11.35	2	3.24	Lys 321	O of C_{24}-OH
			1.15	Ile 468	H of C_{24}-OH

Table 3. Docking results of compounds with HSPG

Compound	S(kcal/mol)	H-bonds	Distance（Å）	Residue	
2a-1	-10.54	-	-	-	-
2a-2	-10.60	2	2.74	His 445	O of C_{12}=O
			2.08	Gln 396	O of C_7=O
2a-3	-8.85	1	2.24	Val 130	O of C_{24}=O
2b-1	-11.45	3	2.31	Arg 104	O of Piperony
			3.25		
			3.22	Val 30	O of Piperony
2b-2	-9.36	1	2.83	Arg 104	O of Piperony
2b-3	-10.28	4	2.15	Arg 104	O of Piperony
			3.16		
			2.83	Val 30	O of Piperony
			2.68	Lys 133	O of C_{12}=O
2c-1	-10.51	-	-	-	-
2c-2	-9.56	-	-	-	-
2c-3	-9.73	1	2.40	Ser 26	O of C_{24}=O
0-1	-11.50	1	2.99	Ser 26	O of C_{24}-OH
0-2	-12.54	1	3.03	Asn 131	H of C_{24}-OH
0-3	-8.43	1	1.93	Lys 133	O of C_{24}=O
Cholic acid	-9.58	3	1.93	Val 130	H of C_{24}-OH
			2.43	Ser 26	H of C_{12}-OH
			2.37	Lys 133	O of C_7-OH
DHCA	-9.31	2	3.51	Arg 25	O of C_7=O
			2.87	Asn 131	O of C_{24}=O

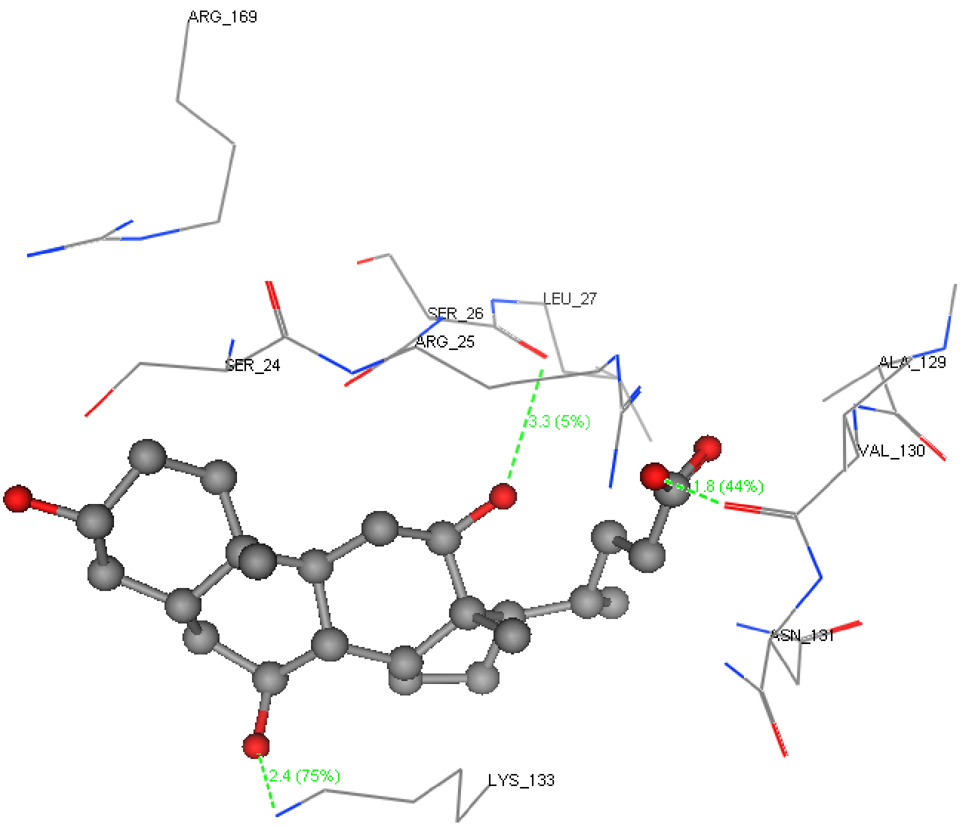

C

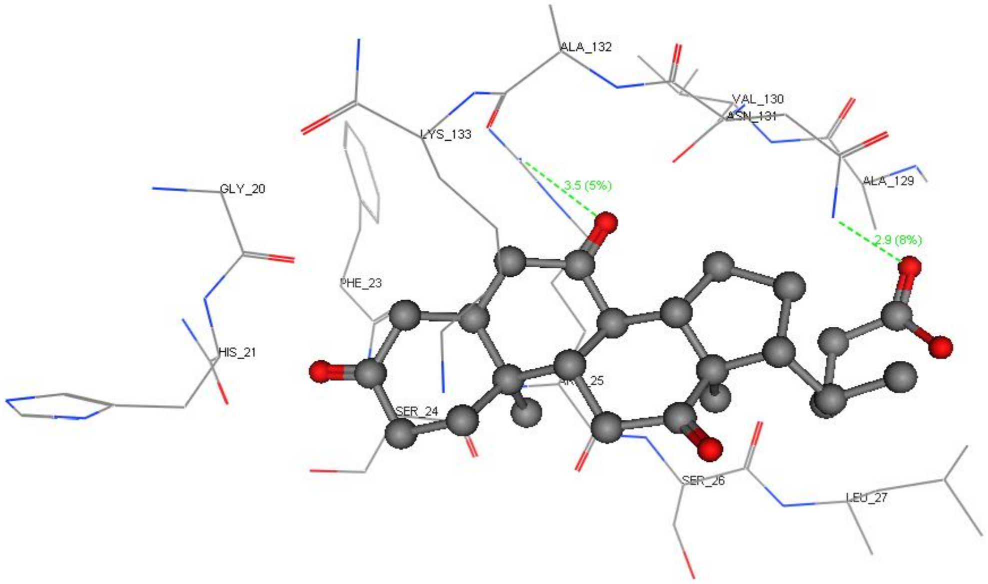

D

Figure 8 Theoretical Binding mode of compound compound cholic acid (C) and dehydrocholic acid (D) in HSPG (pdb: 3sh5) in 3D
